# Impact of COVID-19 Epidemic on Psycho-Oncological Distress in Neuro-Oncological Patients

**DOI:** 10.3390/curroncol30010029

**Published:** 2022-12-26

**Authors:** Franziska Staub-Bartelt, Julia Steinmann, Oliver Radtke, Daniel Hänggi, Michael Sabel, Marion Rapp

**Affiliations:** Department of Neurosurgery, University Hospital Duesseldorf, 40225 Düsseldorf, Germany

**Keywords:** COVID-19, psycho-oncological screening, distress, HADS, brain tumour

## Abstract

Up to 40% of neuro-oncological patients already deal with high levels of distress under conventional circumstances. Due to COVID-19, pandemic hospital visitor rules have been restricted and patients did not receive the same level of supporting caregiver network as before COVID. The aim of the present study was to analyse the impact of the COVID pandemic on the prevalence of distress, anxiety and depression in neuro-oncological patients. Patients admitted for brain tumour surgery were screened regarding distress, anxiety and depression. Furthermore, aspects of patients’ quality of life and clinical data were covered. Retrospectively available data of patients treated pre-pandemic (group A) and throughout the COVID-19 pandemic (group B) were statistically analysed using Chi-square tests and independent-sample *t*-tests, and regression analysis was performed to support statistical findings. Data from 110 patients were available. In all, 48 patients were included pre-COVID-19 and 62 during the COVID-19 pandemic. The authors found no significant difference between pre-COVID-19 prevalence of distress (*p* = 0.112), anxiety (*p* = 0.385) or depression (*p* = 0.084). Regression analyses additionally did not show any significant influence of COVID-19 on the above analysed parameter. Analyses of our cohort’s data could not underline the negative impact of COVID-19 restrictions, shortcuts of professional and remodelled caregiver support on psycho-oncological outcomes.

## 1. Introduction

Stress, also known as psychological distress, results in symptoms such as anxiety and depression and reveals itself when people experience extraordinary emotional pressure. There are external and internal stressors triggering psychological stress, e.g., serious health issues such as cancer diagnosis can also act as a stressor. Levels of distress, anxiety and depression are known to be increased in cancer patients. Up to 35% of cancer patients suffer from distress [1]; additionally, approximately one fourth of cancer patients also indicate depression or depressive symptoms [2].

In the subgroup of neuro-oncological patients, prevalence of distress is even higher with ranges from 38 to 52% [3,4]. Prevalence of depression in brain tumour patients is reported to be around 21% and generally assumed to be higher compared to systemic cancer patients [2,5]. Particularly when hospitalised or in recurrent tumour situations, distress in brain tumour patients increases [6] and special professional support might be required more often. Furthermore, studies revealed the association of increased distress with increased cancer mortality [7] and that distress significantly worsens the outcome in brain tumour patients, especially in patients with high-grade glioma [8,9,10,11].

By releasing stress hormones and glucocorticoids, the body tries to cope with the situation but simultaneously worsens the situation when there is a cancer disease. Studies revealed that in the body’s fight-or-flight reaction with the release of norepinephrine, a stimulation of metastasis [12] can be found and the release of glucocorticoids has negative impacts on tumour progression [13].

Therefore, psycho-oncological screening for diagnosis of increased distress, anxiety or depression is of high relevance, as well as a support system to facilitate a psycho-oncological support.

In the last years, huge effort has been made to install screening instruments for everyday use and cancer centres all over the world have developed professional support systems for their patients.

During the Corona pandemic, treatment capacities were reduced in all areas of medical treatment in order to create treatment capacities for COVID patients and a decrease in the risk of infection. That has led to devastating, effects especially on cancer patients. A systematic review published in 2021 identified 38 categories of delays or interruption of treatments for cancer patients, e.g., chemotherapy, surgery and radiotherapy, and underlined that a delay in cancer treatment without specifying any category was observed in 5–52.6% [14]. In Germany, a prospective questionnaire study targeting 18 Comprehensive Cancer Care Centres (CCCs) after the first wave of the pandemic found that in most of CCCs delays in diagnostic procedures and surgical treatment were not of high concern but restrictions in psycho-oncological care as well as nutrition counselling, physiotherapy and aftercare were reported by nearly all CCCs, and restrictions lasted up to more than 12 weeks [15].

In addition to the elimination of professional supportive systems, contact restrictions that were introduced to the hospitals at the beginning of COVID-19 pandemic led to a reduction in family support as this was only available at restricted visiting hours or, throughout some periods of the pandemic, reduced to digital communication devices.

The COVID-19 pandemic has had an extensive effect on worldwide medical care. With the present study, we aimed to answer the question if COVID-19 had an objectifiable negative impact on psycho-oncological screening parameters, focused on distress, anxiety and depression scores, in neuro-oncological patients. Furthermore, we analyzed the impact if tumour entity or special patients’ situations such as recurrent diagnosis had any influence on the psycho-oncological screening parameters under the light of the COVID-19 pandemic.

## 2. Materials and Methods

In this retrospective single-center analysis (overall screening period March 2019–September 2020), we investigated the impact of the COVID-19 pandemic on the psycho-oncological distress of brain tumour patients. The study was approved by the local ethical committee (Study Number 4087). Reporting of this study aimed to strengthen the reporting of observational studies in the epidemiology (STROBE) guidelines for observational studies (Table S1).

### 2.1. Patients

Inclusion criteria for analysis were as follows: (1) patients age > 18 years with the diagnosis of a brain tumour of any malignancy (2) who were electively admitted for tumour surgery at our neuro-oncological department at University hospital Duesseldorf between (3) January 2019–September 2020.

For further analysis, patients were divided into two subgroups. Subgroup A included patients who were screened before the onset of the first wave of the COVID-19 pandemic between March 2019–September 2019. Subgroup B were patients who underwent screening during the COVID-19 pandemic and federal lockdown measures in Germany between March 2020 and September 2020. Epidemiological information on patients of both groups is summarized in Table 1. As data from 2019 and 2020 were included in the present analysis, the 2016 World Health Organization classification of Tumors of the Central Nervous System [16] was used for neuropathological classification of all included tumors.

#### Screening Assessment

All patients were screened during hospitalisation with the further described tablet-based questionnaire instruments. In case of positive screening results by means of increased scores concerning the used screening items, or in case patients did objectively not have any increased needs but asked for professional support, we introduced the psycho-oncological team to the patients. Patients were instructed in the use of tablets and accompanied during completion by our neuro-oncological team. The following screening questionnaires were used. All of them have been widely discussed in the literature and proved feasibility for screening of psycho-oncological distress [17,18,19,20,21].

### 2.2. National Comprehensive Care Cancer Network, NCCN Distress Thermometer (DT)

The DT is part of the NCCN Guidelines as an easy to administer self-reporting tool with a rating scale ranging from 0 (no distress) to 10 (extreme distress). According to the NCCN guidelines, a DT score of 5 or above is an indicator for distress. Additionally, the DT also contains a list of 40 symptoms representing practical, family, emotional, spiritual-religious and physical concerns. The items in the list provide information about reasons for stress and technical responsibility. There are no systematic data for re-test reliability available as the DT is a visual analogue scale that mostly was used in validation studies with cross-sectional study design.

### 2.3. Hospital Anxiety and Depression Scale (HADS)

The HADS has been established as an effective screening tool for the assessment of anxiety and depression. It is a 14-item self-report questionnaire comprising 7 items used to identify anxiety (HADS-A) and 7 items to identify depression (HADS-D). Each item has a 4-point (0–3) Likert-type scale, and the maximum score on each subscale is 21 points. A cut-off score of >8 is assumed to be optimal concerning sensitivity and specificity in defining anxiety disorders in patients [22,23]. Overall, all 14 items can be subsumed to a reliable scale and be used as a global screening tool for psychiatric disorders [24]. Cronbach’s alpha as a measure for internal reliability for HADS-A is 0.83 (mean) for HADS-D 0.82 (mean).

### 2.4. Health-Related Quality of Life (QoL) Assessment

The EORTC QLQ-C30-BN20 is a disease-specific questionnaire developed by the European Organization for Research and Treatment of Cancer (EORTC) to assess the quality of life of cancer patients comprising the QLQ-BN20, which consists of 20 questions specifically assessing brain tumor-related symptoms. For our analyses, we took a deeper look into the following items: social-functioning, cognitive-functioning, emotional functioning, role functioning, physical functioning and global health status. We chose to include this aspect because previous studies showed that distress and health-related quality of life strongly correlate [25].

### 2.5. Statistical Analyses

Descriptive analyses were performed to investigate patients’ sociodemographic and clinical characteristics. Chi-square tests were performed to assess the impact of COVID on nominal variables (prevalence of distress, anxiety, depression and need for psychosocial support before lockdown vs. during lockdown). Metric variables were analysed by independent-sample *t*-tests (HADS anxiety and depression score, distress thermometer score and QLQ-BN20 scores). Furthermore, multiple linear regression analysis was performed to explore the impact of additional possible factors (physical functioning as indicated by the KPS score, recurrent disease, tumour location, recurrent disease, a.o.) on dependent variables. For correlation of diagnosis and recurrent disease status, the Pearson correlation coefficient was calculated. Statistical significance was set at *p* < 0.05 and all tests were performed two-sided. Computerized analyses were conducted using IBM SPSS Statistics Version 26 (IBM Corp. Released 2019. IBM SPSS Statistics for Windows, Version 26.0. Armonk, NY, USA: IBM Corp).

If data were missing e.g., as described further on in the results Section 3.6 for group B concerning the EORTC QLQ-C30-BN20 questionnaire, we excluded patients from sub-analyses but not from the study as this would have led to significant smaller subgroups and statistical comparison for the main hypothesis could not have been rationally performed.

### 2.6. Informed Consent

The present retrospective study only reports data that were collected at our local neurosurgical ward. Data that are directly accessible for research associates are allowed to be used in scientific context without previous informed consent according to local legislation. Due to anonymization, discerning the identity of individual persons is impossible. Data collection and analyzation included in this study were approved by the local ethical committee, Heinrich-Heine University, Faculty of Medicine, Düsseldorf, Germany (Study number: 4087).

## 3. Results

### 3.1. Cohorts’ Description

Overall, data from 110 patients could be included in the present analyses. In all, 49 patients were male (45%), 61 female (55%). Mean age of the cohort was 59.63 [±14.23 SD]. Overall, 50 patients were diagnosed with high-grade glioma (46%), 9 with low-grade glioma (8%) and 22 patients suffered from cerebral metastases (20%). Moreover, 29 patients were hospitalised due to cerebral lesions of another entity (26%). Mean time from primary diagnosis to data acquisition (screening) was 1.86 years [±3.35 SD].

Patients of group A were screened pre-first wave of the COVID-19 pandemic in Germany between 19 March and 19 September (48 patients, 31 female and 17 male patients with a mean age of 56.88 years [±14.59 SD]). In this group, about one third suffered from high-grade glioma (*n* = 16, 33%), 6 patients from low-grade glioma (13%) and 10 patients were treated due to cerebral metastases (21%). Overall, 33% were hospitalised due to other cerebral entities such as meningioma and pituitary adenoma (*n* = 16).

A total of 60% of the patients in this group received primary diagnoses, 40% recurrent diagnoses mostly in case of high-or-low-grade glioma.

Patients in group B were screened during the first wave of COVID-19 between 20 March–20 September; here, we included 62 patients (30 female, 32 male) with a slightly higher mean age of 61.76 [±13.68 SD]. More than half of the patients were treated due to high-grade glioma (*n* =34, 55%); furthermore, we included 3 patients with low-grade glioma (5%). In all, 12 patients had been diagnosed with cerebral metastases (19%) and 21% were treated because of other entities such as in group A (e.g., meningioma, *n* = 13). Primary and recurrent diagnoses were reported for all 31 patients (50% and 50%).

All patients were either directly treated post-operatively at the neurosurgical normal ward or stayed at the recovery ward. None of the patients were treated at our intensive care unit. Group comparison using independent-sample *t*-tests and Chi-square tests revealed no statistically significant differences between the groups regarding duration of hospitalization, t(108) = −0,40, *p* = 0.688, and physical functioning as indicated by the KPS score χ^2^(2) = 3.76, *p* = 0.152.

### 3.2. Screening Results

In group A, pre-COVID-19 pandemic, 29 patients indicated increased distress (60%). In comparison, 28 patients in group B (during the COVID-19 pandemic) complained about distress (45%). The prevalence of the distress (*p* = 0.112) did not significantly differ between both groups.

The same results could be obtained for prevalence of anxiety and depression. Overall, 10 patients from group A reported increased anxiety (21%) and 6 depression (13%), whereas 9 patients of subgroup B (15 %) reported anxiety and 16 depression (26 %). Therefore, prevalence of anxiety (*p* = 0.385) and depression (*p* = 0.084) did not differ significantly between patients screened pre- or during the COVID-19 pandemic.

### 3.3. Distress Thermometer Scoring (DT Scoring)

**Figure 1:**
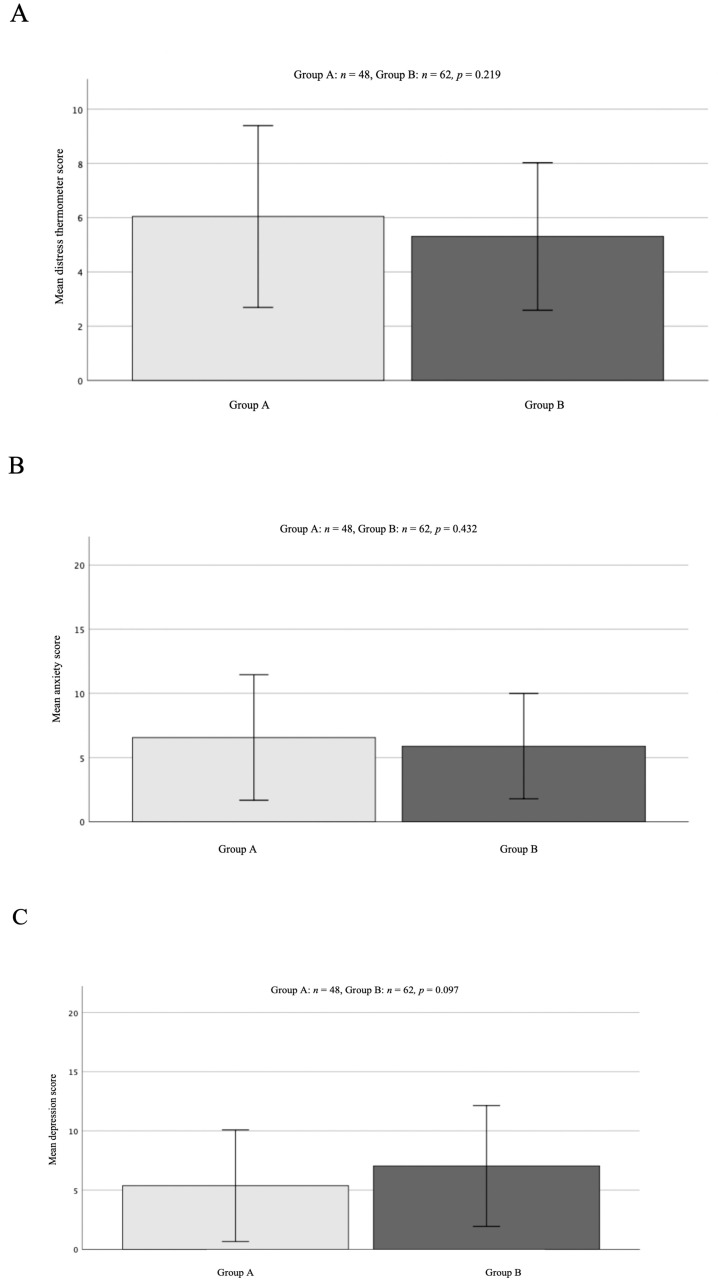
Mean group scores of DT (**A**) and HADS (**B**,**C**) in both cohorts. Mean depression score (**C**) was slightly higher in the COVID-19 cohort; interestingly, DT (**A**) and anxiety (**B**) scores were even higher in the pre-COVID-19 cohort. All results were not significant. *p*-values are stated for each single group comparison.

Regarding the results of the DT assessment, the mean score for group A was 6.04 [±3.35 SD], compared to 5.31 [±2.72 SD] for the patients in group B. Statistical analyses did not reveal a significant difference in the scoring of the distress thermometer in both cohorts (*p* = 0.219, Figure 1A).

Diagnosis regardless of tumour entity had no significant influence on the distress thermometer in both groups (group A *p* = 0.791, group B *p* = 0.567). The same results were obtained when correlating the status of recurrent disease with the DT (group A *p* = 0.972, group B *p* = 0.305).

### 3.4. HADS

The scoring of the anxiety and depression items showed a mean score of 6.56 [±4.89 SD] for anxiety and 5.38 [±4.71 SD] for the depression score in the pre-COVID-19 group (A). In comparison, the mean score in group B was 5.89 [±4.01 SD] for anxiety and 7.04 [±5.01 SD] for depression. Neither of both results reached significance (anxiety group A vs. B *p* = 0.432; depression *p* = 0.097, Figure 1B,C).

We also additionally correlated diagnosis and recurrent disease status in both groups with anxiety and depression scoring, but in line with the DT scoring no significant results could be obtained.

### 3.5. Psycho-Oncological Support

**Figure 2:**
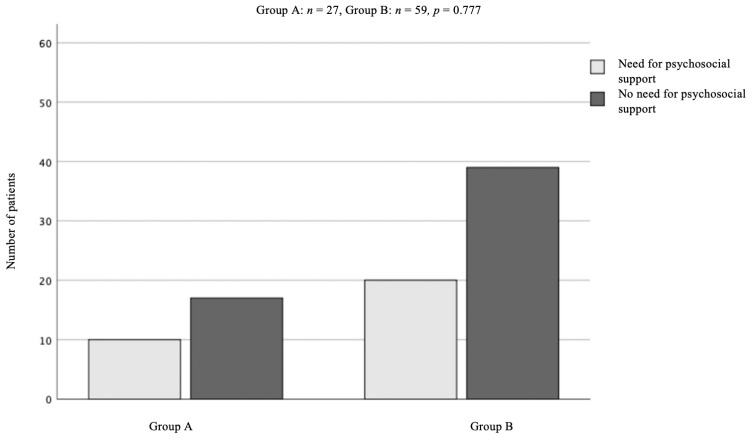
Group comparison of patients who stated they would need psycho-oncological support vs. patients who did not require psycho-oncological support. In group A, we only had 27 of 48, and in group B 59 of 62 datasets available. Regardless of that limited availability, we could not find any statistically significance concerning a hypothesized increased need of professional support under COVID-19 restrictions.

Concerning the subjective need for psycho-oncological support, data from 27 patients in group A were available. Here, 10 patients stated a demand for professional psycho-oncological support during hospital stay (37%). In comparison to group A, data of 59 patients in group B were available of whom 20 patients required professional support (32%). The difference in demand between both groups was not significant (*p* = 0.78) (Figure 2).

### 3.6. EORTC QLQ-C30-BN20 (Assessment of Brain Tumour Related Symptoms and Global Health Status)

We aimed to analyse differences between both subgroups concerning social-functioning, cognitive-functioning, emotional functioning, role functioning, physical functioning and global health status. Neither of the analysed items reached significance when the pre-COVID data and the data obtained under the COVID-19 lockdown were compared. Due to the lack of data in group B, analyses of group B data only comprised 37 patients. The results are summarized in Table 2.

### 3.7. Correlation of Clinical Status DT, Anxiety and Depression Scoring

For clinical evaluation, we used the Karnowski Performance Index (KPS). A KPS of 100–90 was seen in 87 patients pre-op and 85 patients post-op, an index of 80–70 in 21 subjects pre- and post-operatively and a KPS of <70 % was seen in 2 patients pre-op and 4 patients post-op. The KPS did not differ significantly between both timepoints (pre and post-op). The correlation of the KPS and the scoring in the DT and HADS did not show significant correlation between clinical status and one of the analysed scoring parameters neither pre- nor post-op.

## 4. Discussion

The COVID-19 pandemic had and still has enormous influence on patient care in hospital and outpatient department settings. In our study, we aimed to analyze if there had been a significant impact on the psycho-oncological screening outcome in neuro-oncological patients during the first wave of the COVID-19 pandemic due to restrictions in professional patient care and family supportive systems.

In our cohort, prevalence of distress and anxiety did not significantly differ in neuro-oncological patients who were screened pre-COVID and post-COVID first wave. We could observe, even though results were not statistically significant, that mean scores for both screening parameters were higher in the pre-COVID screening group. On the contrary, depression prevalence and mean scores in the depression screening were higher during the COVID-19 screening than before the start of the pandemic in 2020, and the results only narrowly failed to reach significance.

### 4.1. Clinical Implications

In line with our results, an online cross-sectional study, not focusing on patients but standard population with 15.000 participants in Germany published in 2020, showed that prevalence of depression increased under COVID-19 from 5.6 % to 14.3 % [26]. In another meta-analysis focusing on depression and distress in another very special population—health-care workers—analyses of 65 studies revealed the pooled prevalence of depression in 21.7% and 22.1% for anxiety [27]. In comparison, we observed a depression prevalence of 26% and 15% for anxiety in our neuro-oncological patient’s cohort. These results underline that the cohorts not involved in the medical environment either professional or as a patient tend to have lower levels of depression under COVID-19 and vice versa, but also in the general population prevalence of depression increased. Recently, Obispo-Portero et al. reported their study results of 401 cancer patients with an overall higher prevalence of depression and anxiety under COVID-19 in patients with advanced cancer diagnoses and additionally found that younger age <65 years was a risk factor for higher distress levels but that the tumor stage did not have any influence on distress levels during the pandemic [28]. Already in 2010, in times when a worldwide pandemic seemed to be a historical incident, Hinz et al. reported that cancer patients are generally at higher risk for depression and anxiety compared to the general population; and particularly in younger patients, prevalence was nearly twice as high in their analysis. On the contrary, the tumor stage also did not have any significant influence comparable to the findings of Obispo-Portero et al. [29].

In our cohort age, recurrent diagnosis and entity of diagnosis, either malignant or benign, had no influence on the psycho-oncological outcome of the patients independently of screening timepoint. However, we actually had expected some higher distress levels in patients with recurrent diagnosis as there are specific timepoints reported in the literature in neuro-oncological patients where an increased distress was observed, especially during hospitalisation as well as at the time point of tumour recurrence [30], which might be due to various general apprehension when diagnosed with recurrent cancer.

In our additional analysis of specific items of the quality-of-life questionnaire, we found that there was no statistical difference in social-functioning, cognitive-functioning, emotional functioning, role functioning, physical functioning and global health status in both of our subgroups; and interestingly, under COVID-19, patients did not ask for professional support more often under COVID-19.

Our data show that circumstances that came along with the first wave of COVID-19 did not have significantly negative effects on the psycho-oncological outcome of our neuro-oncological patients.

In consideration of what we have learned from recent studies, these results were somehow surprising to the authors. We know that neuro-oncological patients tend to have higher distress levels when they are hospitalized or in recurrent situations. We hypothesized that under COVID-19 these effects would have been even more increased. On the contrary, our cohort did not show significant differences in both groups and recurrent diagnoses did also not correlate with higher scoring results.

There are some points that might have contributed to that fact the authors would like to point out.

Firstly, patients who are diagnosed with brain tumors are burdened due to the diagnose to such an extent and focused so much on that mostly felt life-threatening incident that further external influences might not play such a great role to them. Taking that into consideration, maybe the patients’ relatives were the ones who suffered from COVID-19 restrictions even more due to strict visiting hours or even visiting interdictions and the subjective feeling to be even more helpless due to these special circumstances.

Another aspect of the patient relative relationship is that we have learned from different studies that used Fear of Progression Questionnaires (FoP-Q) that both parts tend to the mutual burden each other. One can hypothesize that the more distant relationship under the COVID-19 pandemic might have a positive influence on some patients’ disease and therapy perception.

On the contrary, the restrictions of the first wave of the COVID-19 pandemic unarguably have led to enormous changes for hospitalized patients, particularly when it comes to availability of family support systems, especially for those patients who strongly depend on family care structures. In our department, we aimed to connect closely with patients’ families via daily phone calls and encouraged patients and families to make use of multi-media devices to keep up their daily contact.

The use of these tools to overcome the visiting restrictions as well as the engagement of additional urgent professional support whenever special needs were diagnosed might have contributed to the reported results, which we evaluate as positive findings under the given circumstances.

### 4.2. Limitations

Regarding sensitivity and specificity of the used measures for the diagnosis of certain psychiatric diseases such as anxiety and depression, there are different cut-off values published and used in the literature. That might lead to different numbers of diagnoses in patient cohorts when different cut-offs are used. Nevertheless, in the present study, the questionnaires were only used as screening instruments not as a diagnostic tool, and there is a wide range of published data agreeing about feasibility as screening instruments.

At last, concerning the requirement of psycho-oncological support and EORTC-C30-BN20 datasets, we can only report analysis of limited datasets as there were only 27 instead of 48 datasets in group A and only 59 instead of 62 patients’ datasets available for group B (psycho-oncological support) and only 37 instead of 62 datasets in the EORTC-Bn20 screening for group B. A further limitation is the arguably very specific and small subgroup of neuro-oncological patients who also split into further subgroups of benign and malign diagnoses. Nevertheless, as we could show that the entity of diagnosis had no significant influence, our results can be conveyed to the subgroup of neuro-oncological patients. However, the small number of patients could also have contributed to our findings reported above.

## 5. Conclusions

Here, we present data of a very special group of cancer patients. In the present cohort of neuro-oncological patients, the impact of COVID-19 restrictions and shortcuts of professional and remodelled caregiver support did not have a significant influence on psycho-oncological outcome in terms of distress, anxiety or depression in the cohort. Our data show that a dedicated communication and a very close patient and patients’ family guidance might help to overcome gaps in professional psycho-oncological support even in very special situations such as we have experienced in the past two years.

## Figures and Tables

**Table 1:** Descriptive epidemiologic data of patient cohort.

	Pre-COVID-19 Pandemic (*n* = 48)	During COVID-19 Pandemic (*n* = 62)
AGE (y)		
mean	56.88 [SD ± 14.59]	61.76 [SD ± 13.68]
range	21–83	29–86
GENDER		
female	31	30
male	17	32
DIAGNOSIS		
High-grade Glioma (WHO III + IV)	16	34
Low-grade glioma (WHO I + II)	6	3
Cerebral metastases	10	12
Other	16	13
PRIMARY DIAGNOSES	29	32
KPS (%)		
100–90	87	85
80–70	21	21
<70	2	4

**Table 2:** Results from the EORTC QLQ-C30-BN20 questionnaire. Neither of the analysed items reached significance when mean scores of pre-COVID and during COVID screening were compared. However, in group B we only had 37 of 62 datasets available, which might limit confidence of results.

	Pre-COVID-19 Pandemic (*n* = 48)	During COVID-19 Pandemic (*n* = 37)	*p* =
SOCIAL-FUNCTIONING mean score	64.35 [SD ± 35.22]	76.81 [SD ± 29.90]	0.266
COGNITIVE-FUNCTIONING mean score	61.15 [SD ± 30.49]	65.46 [SD ± 25.81]	0.082
EMOTIONAL-FUNCTIONING mean score	56.33 [SD ± 30.83]	55.95 [SD ± 28.78]	0.994
ROLE FUNCTIONING mean score	55.71 [SD ± 39.76]	47.51 [SD ± 38.12]	0.631
PHYSICAL FUNCTIONING mean score	61.98 [SD ± 32.77]	49.03 [SD ± 34.09]	0.698
GLOBAL HEALTH-STATUS mean score	45.52 [SD ± 28.40]	45.84 [SD ± 25.35]	0.544

## Data Availability

Data are available on request from corresponding author.

## Supplementary Materials

The following supporting information can be downloaded at: https://www.mdpi.com/article/10.3390/curroncol30010029/s1, Table S1: STROBE Statement—checklist of items that should be included in reports of observational studies.
